# Toward Continuous Molecular Testing Using Gold-Coated Threads as Multi-Target Electrochemical Biosensors

**DOI:** 10.3390/bios13090844

**Published:** 2023-08-25

**Authors:** Martin Hanze, Shirin Khaliliazar, Pedro Réu, Anna Toldrà, Mahiar M. Hamedi

**Affiliations:** Department of Fibre and Polymer Technology, School of Engineering Sciences in Chemistry, Biotechnology and Health, KTH Royal Institute of Technology, Teknikringen 56, 10044 Stockholm, Sweden

**Keywords:** metal-coated threads, self-assembled monolayers, isothermal DNA amplification, roll-to-roll, sandwich hybridization assay, chronoamperometry

## Abstract

Analytical systems based on isothermal nucleic acid amplification tests (NAATs) paired with electroanalytical detection enable cost-effective, sensitive, and specific digital pathogen detection for various in situ applications such as point-of-care medical diagnostics, food safety monitoring, and environmental surveillance. Self-assembled monolayers (SAMs) on gold surfaces are reliable platforms for electroanalytical DNA biosensors. However, the lack of automation and scalability often limits traditional chip-based systems. To address these challenges, we propose a continuous thread-based device that enables multiple electrochemical readings on a functionalized working electrode Au thread with a single connection point. We demonstrate the possibility of rolling the thread on a spool, which enables easy manipulation in a roll-to-roll architecture for high-throughput applications. As a proof of concept, we have demonstrated the detection of recombinase polymerase amplification (RPA) isothermally amplified DNA from the two toxic microalgae species *Ostreopsis* cf. *ovata* and *Ostreopsis* cf. *siamensis* by performing a sandwich hybridization assay (SHA) with electrochemical readout.

## 1. Introduction

Electroanalytical systems provide the opportunity to quickly, inexpensively, and specifically identify pathogens [1,2], and their electronic readout mode enables direct digital readout without the need for optical instrumentation (e.g., gel-doc or qPCR thermocycler) [3].

The use of electroanalytical systems for nucleic acid amplification tests (NAATs) enables reliable, sensitive, and specific tools for electronic detection of pathogens. PCR is the gold standard nucleic acid amplification technique, but it is difficult to integrate into compact devices, as it requires advanced thermal cyclers. To this end, several isothermal amplification methods have been developed as an alternative to PCR [4,5], such as nucleic acid sequence-based amplification (NASBA) [6,7], loop-mediated isothermal amplification (LAMP) [8,9,10], strand displacement amplification [11,12], helicase-dependent amplification (HDA) [13], rolling circle amplification (RCA) [14,15], and recombinase polymerase amplification (RPA) [16,17]. Among them, RPA is particularly attractive because it is faster (20–40 min), requires lower temperatures (37–42 °C) [18,19,20], and has been shown to amplify down to a single copy [4].

The most reliable electroanalytical DNA biosensors are built on self-assembled monolayers (SAM) [21,22]. This has been demonstrated, for example, by Carr et al., who detected DNA methylation with a DNA biosensor using oligonucleotide hybridization probes adsorbed to an 11-mercaptoundecanoic acid SAM on a Au surface [23]. Likewise, Chen et al. were able to detect methylated DNA by combining oligonucleotide SAMs on both magnetic Au nanoparticles and Au foil surfaces [24]. It has also been demonstrated in our work on detecting RPA amplification products with SAMs of DNA capture probes on Au threads [1,2,25] and printed circuit board Au surfaces [25,26] with stem-loop probes and sandwich hybridization assay (SHA).

Electroanalytical systems are usually fabricated on individual chip-like platforms [27,28,29,30,31,32,33], which are typically not well-suited for automation. One attractive architecture for automation is a roll-to-roll system where molecular diagnostics based on electrochemical detection would be integrated. Safavied et al. previously described one such system relying on LAMP for the detection of pathogenic bacteria [34]. However, this system was limited by the utilization of chips that, although connected by a flexible ribbon for roll-to-roll movement, still required individual connections, making its upscaling and automation difficult.

Here, we present a method that overcomes both the scaling and connection issues for biosensing using a continuous thread-based device capable of embedding multiple electrochemical bioassays with readout at a single connection point. The thread-based device is based on self-assembled DNA monolayers and can perform sequential measurements on different parts of the same continuous thread, which can be rolled, in contrast to our earlier work on these Au threads, which were limited to a single measurement per device and had a chip-like architecture unsuited for automation [25]. As a proof of concept, functionalized off-the-shelf gold plasma-coated threads were rolled on a spool to showcase a roll-to-roll architecture and, following an SHA, detect RPA-amplified DNA of the microalgal species *Ostreopsis* cf. *ovata* and *Ostreopsis* cf. *siamensis*, known to cause toxic blooms associated with skin and respiratory problems in humans and mortality of marine organisms [35,36].

## 2. Materials and Methods

### 2.1. Materials

Monobasic potassium phosphate (KH_2_PO_4_), dibasic potassium phosphate (K_2_HPO_4_), skimmed milk powder, Tween 20, nuclease-free water, and Advanced PAP (A-PAP) Pen were purchased from Sigma-Aldrich (Solna, Sweden). Pierce™ TMB enzymatic substrate for horseradish peroxidase (HRP) (containing 0.4 g/L 3,3′,5,5′-tetramethylbenzidine (TMB) and 0.02% hydrogen peroxide) was bought from Fisher Scientific (Gothenburg, Sweden). TwistAmp Basic RPA kit was purchased from TwistDX Limited (Maidenhead, UK). QIAquick PCR purification kit was purchased from QIAGEN (Stockholm, Sweden). Custom DNA oligonucleotides were synthesized by Biomers (Ulm, Germany) (see Table S1 for the oligonucleotide sequences). Plasma-coated gold and silver multifilament threads were purchased from Swicofil (Emmen, Switzerland) (see Table S2 for the specifications of the threads).

### 2.2. Device Assembly

A pattern of four reaction regions was marked out with a normal marker pen on a standard microscope glass slide by tracing a printed template. On the opposite side of the glass slide, the outer edges and in-between regions of the reaction regions were marked with a PAP pen, thus defining hydrophilic reaction regions surrounded by a hydrophobic barrier. The thread support structures were made from 100 µm thick laminate plastic that was cut based on a digital template with a ScanNcut CM900 cutter machine (Brothers, Sweden). Four layers of cut laminate plastic were aligned around and in between the wells. Additionally, two rectangular pieces of laminate plastic were added to one of the short ends of the glass slide to form a thin extension to help with electrode connection during electrochemical readings. The plastic layers were laminated to the glass slide using an E-15S hot press (SkilteProduktion, Nørresundby, Denmark) at 120 °C for 1 min. Threads were attached to the device using strips of double-sided tape on either side of and between the wells on the plastic support structure.

### 2.3. Functionalization of Au Threads

**Figure 1:**
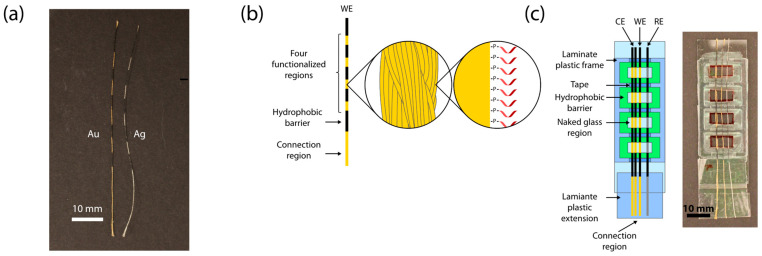
Schematic of functionalized threads and device: (**a**) A photograph of patterned Au and Ag threads. (**b**) Schematic of patterned, SAM functionalized Au thread used as the working electrode. (**c**) Schematic of the glass slide device and its components. The right side shows a photograph of the completed device for comparison. WE = functionalized Au thread working electrode, CE = two Au threads acting as a counter electrode, RE = Ag thread pseudo-reference electrode.

Four reaction areas with a 5 mm length were defined by adding nail polish to the single Au thread (approximately 7 cm), following a printed template (Figure 1A). To label the defined areas, the threads were aligned and attached to the glass device. A 100 µL volume of capture probe (Table S1) solution (500 nM, in PBS 1X) was directly added to the wells for overnight incubation at 4 °C (Figure 1B). Following the overnight incubation, the capture probe solution was removed from the wells by careful absorption with a tissue. The modified areas were rinsed by pipetting 120 µL PBS-Tween-20 solution (100 mM phosphate, 150 mM NaCl, pH 7.2, 0.05% Tween-20) followed by 200 µL PBS (100 mM phosphate, 150 mM NaCl, pH 7.2) to each well with intermediate removal through absorption. The wells were used immediately or filled with 100 µL PBS buffer to keep the threads wet for up to 1 h before the devices were used for the SHA and electrochemical detection.

### 2.4. Rolling Threads on Spool

**Figure 2:**
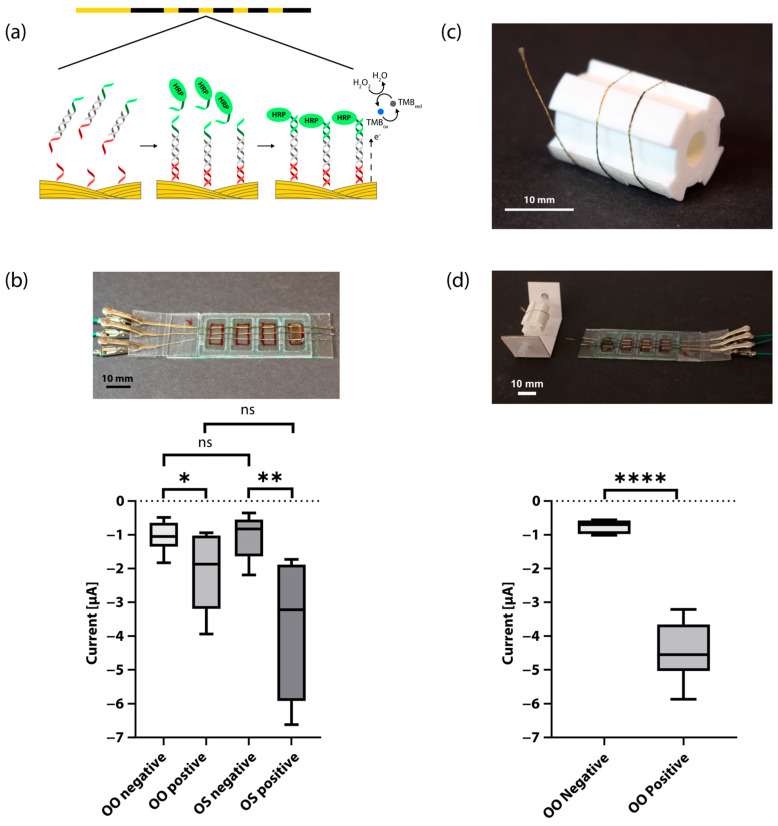
Results for the electrochemical biosensor: (**a**) Schematic overview of the sandwich hybridization assay. The amplified DNA product with 5′ and 3′ single-stranded tails hybridizes with the capture probe and subsequently the HRP-tagged oligonucleotide reporter probe. HRP catalyzes a reaction where the TMB substrate is oxidized with hydrogen peroxide, which is reduced back during chronoamperometric measurements. (**b**) Results of chronoamperometry with two targets. The upper half shows a photograph of the finished glass slide device with mounted electrode threads, clips connected to the electrode threads, and 100 µL liquid (water as a demonstration) added to the fourth region from the connection region. The box plot at the bottom shows the average current of chronoamperometry for positive and negative samples of both *O*. cf. *ovata* (OO) and *O*. cf. *siamensis* (OS). There was a significant difference in current between negative (mean M = −1.037 µA, standard deviation SD = 0.4143 µA) and positive (M = −2.063 µA, SD = 1.061 µA) OO samples (*n* = 7, *p* = 0.032), as well as between negative (M = −1.067 µA, SD = 0.6069 µA) and positive (M = −3.716 µA, SD = µA) OS samples (*n* = 7, *p* = 0.0028). Legend: ns means no significant difference, the asterisks (*) mean significant difference (one: *p* ≤ 0.05, two: *p* ≤ 0.01) (**c**) Photograph of a patterned, functionalized working electrode Au thread rolled onto a spool with a 4-pin cogwheel-shaped cross-section with functionalized regions aligned with the grooves. The thread is longer than those typically used in experiments to be able to roll it more than two revolutions around the spool for demonstration purposes. (**d**) Chronoamperometric detection on threads that have been rolled onto a spool after functionalization. The photograph on the top shows a functionalized Au thread that has been rolled out from a spool onto a glass slide device. The thread is longer than those typically used in the experiments for demonstration purposes. The box plots at the bottom show the average current for positive and negative samples of *O*. cf. *ovata* (OO). There was a significant difference in current between negative (M = −0.7537 µA, SD = 0.1765 µA) and positive (M = −4.460 µA, SD = 0.8264 µA) samples (*n* = 6, *p* < 0.0001). Legend: ns means no significant difference, the asterisks (*) mean significant difference (four: *p* ≤ 0.0001).

The spool (Figure 2C) was designed in AutoCAD 2022 software (AutoDesk, San Francisco, CA, USA) and is a cylinder with a cogwheel-shaped cross-section with four pins and four grooves of equal arc length (octants). In experiments when th e working electrode thread was rolled onto the spool, the functionalized thread with four functionalized regions was carefully rolled with one revolution onto the spool with the functionalized regions aligned with the grooves before the attachment to the glass slide device.

### 2.5. DNA Amplification

An RPA reaction was used to isothermally amplify DNA using the TwistAmp Basic Kit (TwistDx Ltd., Cambridge, UK). The RPA master mix preparation was based on a previously described work [37] and was prepared as follows: for each reaction, 2.4 μL of each of the two primers (10 μM), 14.75 μL of the supplied rehydration buffer, 22.95 μL nuclease-free water, and ½ enzyme pellet were mixed. The pellets were allowed to rehydrate in the master mix for 4 min. Then, 42.5 µL of the master mix was transferred to each reaction tube. A 5 μL template (1 pM synthetic DNA for positive samples or nuclease-free water for negative samples) was added to each tube. The RPA reactions were initiated simultaneously by adding 2.5 μL of the provided MgOAc solution to the tube lids and then centrifuging the tubes together. The reaction mixtures were then mixed by inverting the tubes 10 times and were incubated at 37 °C for 30 min in a block heater. After 4 min from the start of the incubation, the tubes were inverted vigorously 8–10 times, spun down, and placed back in the thermal heater for the remaining reaction time. The reaction was stopped by putting the tubes in a −20 °C freezer. The RPA products were purified using the QIAquick kit following the manufacturer’s instructions. Purified RPA product was stored at −20 °C until further use.

### 2.6. Gel Electrophoresis

All purified RPA products were screened for successful amplification (presence of DNA amplicons of the expected length and absence of other bands) in positive samples and the absence of amplifications in negative samples through gel electrophoresis before further use. For each sample, 5 µL of RPA product was mixed with 1 µL of loading buffer, and 5 µL of the mixture was loaded on a 2% *w/v* agarose gel containing GelRed stain. Amplicon base pair length was estimated with a DNA ladder. The gel was imaged with a ChemiDoc XRS+ (BioRad, Solna, Sweden).

### 2.7. Sandwich Hybridization Assay and Electrochemical Detection

For the SHA, 45 µL purified RPA product was mixed with 55 µL PBS buffer and was loaded on the functionalized regions on the threads and incubated for 30 min to allow hybridization to the capture probes. After washing (same procedure as after functionalization, see Section 2.3 “Functionalization of Au threads”), 100 µL of 20 nM HRP-tagged capture probe in 2% *w/v* skim milk in PBS was loaded on the test regions and incubated for 30 min to allow hybridization to the amplicons, followed by another washing step. At this stage, counter and pseudo-reference electrode threads were added to the device. Chronoamperometric measurements were performed using an Autolab PGSTAT204N with MUX 16 module (Metrohm Autolab, Bromma, Sweden) with the accompanying NOVA 1.11 software package. First, 100 µL of an enzymatic substrate mixture containing equal volumes of H_2_O_2_ and TMB was loaded on each well sequentially. After allowing the mixture to react for 3 min, chronoamperometric measurements were carried out by applying a reducing potential of −0.2 V for 10 s and then reading the current output at the 1 s mark. The reporter probe mixture and loaded devices were kept in the dark whenever possible during the incubation steps and assay. The current data were analyzed with unpaired, two-tailed Student’s t-test in Prism 10 (GraphPad, Boston, MA, USA) with a significance level α = 0.05.

### 2.8. Electrical Signal Comparison

The modified hybridization assay to compare signal strength between two reaction regions was performed by functionalizing the working Au electrode thread with a capture probe complementary to the HRP-tagged reporter probe (Table S1), thus obtaining a response signal that was independent of amplification yield. Washing steps and chronoamperometry readings were performed the same way as described in Section 2.7 “Sandwich hybridization assay and electrochemical detection”. This modified assay was used to assess if there was a potential drop across a longer distance of the thread electrodes by attaching two glass devices to the same set of Au and Ag electrode threads with a space of 50.0 cm between the functionalized region of the working electrode closest to the connectors and the second functionalized region. These sample groups were analyzed with paired, two-tailed Student’s t-test with a significance level α = 0.05.

## 3. Results and Discussion

Self-assembled monolayers (SAMs) are among the most reliable methods to perform electroanalytical DNA tests with high specificity. The most common integration of SAM relies on a discrete device (e.g., chips) with micropatterned electrodes, combined with other microfluidic systems [21].

Here, we utilized a continuous gold-coated thread to perform SAM-based DNA electroanalysis at multiple detection sites without significant cross-interference. While individual chips are hard to handle by a machine, a thread can easily be manipulated simply by rolling. This is advantageous both during the manufacturing of the surface, which must be modified, and during utilization within the actual detection device. These Au threads have been used in our previous work for the electrochemical detection of isothermally amplified DNA with stem-loop probes [1,2] and SHA [25] strategies, and the Au threads have been integrated into woven textile [2,38], stitched textile [39] and paper-based [25] devices. We have shown that Au threads can be functionalized off-the-shelf without gold electroplating or any laborious and risky washing steps like washing with Piranha solution, which Au electrode surfaces often need. Earlier works on these Au threads have been limited to one measurement per device; however, many applications require the inclusion of additional test controls, multiple targets, and potentially replicated measurements to ensure the reliability of the results. Here, we show that a single thread can be used for multiple measurements and different targets and show the potential of rolling functionalized threads for further applications like automated devices with a roll-to-roll architecture. By stabilizing the oligonucleotide SAMs and protecting them from mechanical damage, we believe more complex manipulation of the threads could be possible.

We functionalized a single continuous off-the-shelf Au thread in four distinct regions (Figure 1A) by attaching it to a simple device (Figure 1C and Figure 2B), which was made from a glass slide with four regions framed by a raised structure made from laminate plastic. We incubated the Au thread overnight with thiol-tagged single-stranded oligonucleotides that are designed to act as capture probes, spontaneously forming a monolayer due to the interactions between the thiol group and the Au surface [40,41,42,43]. The functionalized Au thread acted as a working electrode, two Au threads acted as the counter electrode, and an Ag thread acted as the pseudo-reference electrode (Figure 1C). We amplified the DNA of the microalgae species *O.* cf. *ovata* and *O.* cf. *siamensis* using tailed primers and RPA (Figure S1), and purified and screened for successful amplification with agarose gel electrophoresis (Figure S2). We used positive controls with a template concentration of 1 pM, which corresponds to 1500 microalgal cells/reaction. Based on the fact that one cell of *O.* cf. *ovata* has 2137 ribosomal DNA copies per cell [44], the concentration used may allow quantifications below the current alarm thresholds proposed for *Ostreopsis* cells (10,000–30,000 cells per liter of seawater) [45]. We also used negative controls in which the template had been replaced with nuclease-free water. Although we showed a qualitative biosensor, quantitative measurements could also be performed, as already demonstrated in our previous work using the same Au threads and detection strategy, in which we reached an LOD of 0.06 pM (which corresponds to 85 microalgal cells/reaction) [36]. This indicates that lower limits can be achieved if needed. The only other electrochemical roll-to-roll system that has been reported detected pathogenic bacteria with detection limits of 30 CFU/mL for *Escherichia coli* and 200 CFU/mL for *Staphylococcus aureus* [34].

We performed a SHA [46,47,48] using the capture probes immobilized on the Au thread and oligonucleotide-tagged HRP reporter probes (Figure 2A). In this assay, HRP catalyzes a reaction in the presence of 3,3′,5,5′ tetramethylbenzidine (TMB) and hydrogen peroxide in which TMB is oxidized, allowing an electrochemical analysis with chronoamperometry at −0.2 V. This voltage was chosen based on initial investigation with circular voltammetry on Au threads in TMB and H_2_O_2_ (Figure S5). Readout was performed at 1 s in the region with the highest discrimination between positive and negative samples. Figure S3 shows representative chronoamperometry graphs measured for a positive and a negative RPA-amplified DNA sample of the *O.* cf *ovata* algae. A benefit of utilizing amperometric readout is its ease of implementation with low-cost off-the-shelf electronics, as demonstrated in our earlier work on portable potentiostats [26].

For each device with four reaction regions, we detected RPA-amplified DNA of both *O.* cf *ovata* and *O.* cf. *siemensis* (one positive and one negative sample of each, *n* = 7) on the same thread (Figure 2B). In this case, the thread had not been rolled on a spool beforehand. There was a significant difference in current between negative and positive samples for both microalgae species. With this, we demonstrated the possibility of including multiple targets in the same assay panel.

To demonstrate the possibility of a roll-to-roll architecture for high-throughput applications, we also rolled the working Au electrode thread onto a spool with a 4-pin cogwheel-shaped cross section after functionalization (Figure 2C) and unrolled it onto a new device before the start of the SHA (Figure 2D). In this case, we detected two positive and two negative samples of *O.* cf *ovata* per each device (*n* = 6). Again, there was a significant difference in current between negative and positive samples. This shows that it is possible to roll up functionalized threads for storage.

**Figure 3:**
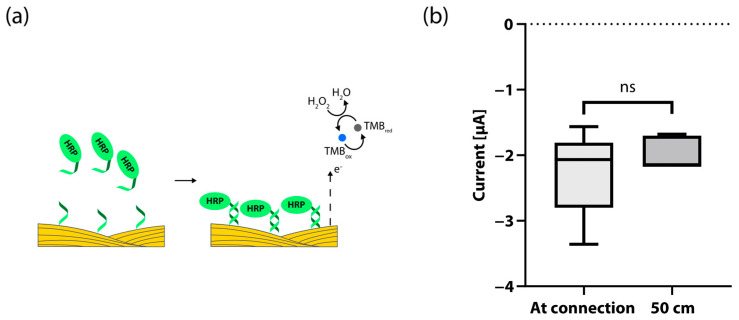
Device characterization: (**a**) Schematic of the alternative approach used to compare signal strength unaffected by variation in amplification yield. The capture probes used bind directly to the reporter probe. (**b**) Test for signal drop over a distance of 50.0 cm. Box plots show average chronoamperometric current after 1 s for reaction regions positioned 41 mm and 541 mm from the connection points. There was no significant difference (*n* = 5, *p* = 0.3105) in current between the measured region closest to the connector (M = −2.257 µA, SD = 0.5945 µA) and the one 50.0 cm down the thread (M = −1.979 µA, SD = 0.2276 µA). Legend: ns means no significant difference.

In order to perform multiple runs of an assay panel, the threads need to be extended accordingly. A loss of signal could be expected with measurements far from the connection region, due to intrinsic properties like resistivity or other factors in the threads. To test for possible signal losses, we used a modified hybridization assay to compare just the signal strength by functionalizing the working electrode threads with oligonucleotide single-stranded capture probes that bind directly to the HRP-tagged reporter probe, thus eliminating variance occurring from varying amplification yield (Figure 3A). We compared the current response of two functionalized regions 50.0 cm apart on the same thread (see Figure S4 for a schematic of the experimental setup and Figure 3B for plotted data). As expected, there was no significant difference in current between the measured region closest to the connector and the one 50.0 cm down the thread. This undetectable loss of signal strength is in accordance with the reported resistivity values of the threads [38]. These results show that threads can be used as the substrate for multiple connected biosensors that are first rolled up and then used with distances reaching into meter scales for analytical devices.

## 4. Conclusions

We have shown that off-the-shelf Au threads functionalized with DNA SAMs can act as multi-target, multi-assay biosensors that can be rolled on and off spools without signal loss. This proof-of-concept work shows that threads can pave the way for automated roll-to-roll electronic molecular diagnostic biosensors. Our functionalized thread-based electrodes could be stored while rolled on a spool and subsequently be rolled out before use. While our device has four discrete reaction regions, the dimensions of the device and spool could easily be adjusted to accommodate assay panels that need a higher number of reactions, to a reasonable extent. Furthermore, multiple runs of the assay could potentially be stored on the same spool as long as the thread does not cross itself. This kind of device could contribute to early detection and monitoring with potential applications including continuous environmental surveillance, medical diagnostics, or food safety monitoring. Future development should aim at integrating these thread-based biosensors into automated roll-to-roll microfluidic systems with integrated electroanalytical compact devices [26] and further cost-reduction, for example, by minimizing the use of enzymes.

## Data Availability

The data presented in this study are available on request from the corresponding author.

## Supplementary Materials

The following supporting information can be downloaded at: https://www.mdpi.com/article/10.3390/bios13090844/s1, Table S1: List of oligonucleotide sequences and their respective modifications; Table S2: Threads used in this study and their characteristics; Figure S1: Steps of the RPA cycle with tailed primers; Figure S2: A characteristic example of an agarose gel electrophoresis of purified RPA product; Figure S3: Representative plot of chronoamperometric measurement; Figure S4: Schematic of two devices connected by long threads with two reaction regions spaced 50.0 cm apart; Figure S5: CV of Au threads in TMB and H_2_O_2_.
