# Supplementary material for: Toward Continuous Molecular Testing Using Gold-Coated Threads as Multi-Target Electrochemical Biosensors
Source: Biosensors (Basel). 2023 Aug 25;13(9):844. doi: 10.3390/bios13090844 (PMC10526339; doi:10.3390/bios13090844)
Supplement: Supplementary file 1 [file biosensors-13-00844-s001.zip › biosensors-2509368-supplementary.pdf]

Supporting Information

Towards Continuous Molecular Testing Using Gold-Coated Threads as Multi-Target Electrochemical Biosensors

Martin Hanze, Shirin Khaliliazar, Pedro Réu, Anna Toldrà, Mahiar M. Hamedi**

Department of Fibre and Polymer Technology, School of Engineering Sciences in Chemistry, Biotechnology and Health, KTH Royal Institute of Technology, Teknikringen 56, 10044, Stockholm, Sweden.

Table S1. List of oligonucleotide sequences and their respective modifications (underlined).

**Name**	**Sequence (5′-3′)**
O. cf. *ovata* FwP with tail	gtt ttc cca gtc acg ac-C3-aca atg ctc atg cca atg atg ctt gg
O. cf. *siamensis* FwP with tail	att acg acg aac tca atg aa-C3-tga gtt tgt gtg tat ctt gca cat gc
Ostreopsis spp. RvP with tail	tgt aaa acg acg gcc agt-C3-gca wtt ggc tgc act ctt cat aty gt
O. cf. *ovata* thiolated capture probe	gtc gtg act ggg aaa act ttt ttt ttt ttt tt-C3-SH
O. cf. *siamensis* thiolated capture probe	ttc att gag ttc gtc gta att ttt ttt ttt ttt tt-C3-SH
Reporter probe-binding capture probe	ttt ttt ttt ttt ttt tgt aaa acg acg gcc agt
HRP-tagged reporter probe	HRP-act ggc cgt cgt ttt aca
O. cf. *ovata* target	aca atg ctc atg cca atg atg ctt ggt ggc atg cac ctt gtt agt tgt agc atg aca gct tga tac tta tct aaa cgc ttt cat caa ctg tct tct gac agc aat gaa tgc atc aat tca aaa caa tat gaa gag tgc agc caa atg c
O. cf. *siamensis* target	tga gtt tgt gtg tat ctt gca cat gca tgt aaa cac ata tgc ttc act ata agt ttg cat a gtg tgc tgt cct tga ctt cat ttt agt tgc agc aat gaa tgc atc aat tca aaa cga tat gaa gag tgc agc caa atg c

Table S2. Threads used in this study and their characteristics.

**Thread Type**	**Yarn Number**	**Filament. No**	**Diameter (μm)**	**Supplier**
Gold Plasma coated yarn Polyester FDY, high bright (1.1 mg/m Au)	dtex 125/f36/2	72	246 ± 20	Swicofil AG
Silver Plasma coated yarn Polyester FDY, high bright (3.9 mg/m Ag)	dtex 125/f36/2	72	185 ± 19	Swicofil AG

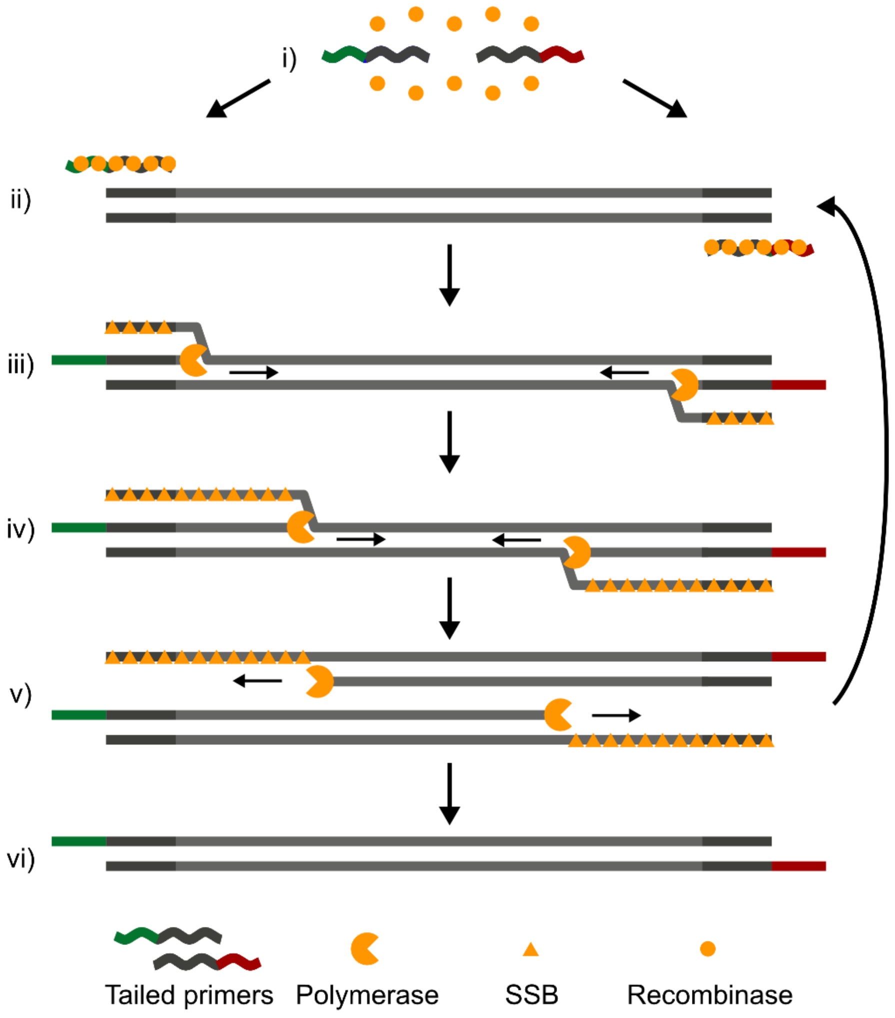

Figure S1. Steps of the RPA cycle with tailed primers: i) The tailed oligonucleotide primers associate with recombinase in the reaction mix. ii) Recombinase/oligonucleotide complexes target the homologous regions on the double-stranded template DNA. iii) Strand exchange between the primers and template strands forms D-loops which are stabilized by single-stranded DNA binding (SSB) proteins. iv) Polymerase initiates DNA synthesis, extending from the primers. v) Parental strands separate and polymerase continues the extension of the synthesized strands until complete duplexes are formed and the cycle repeats. Note that there is a spacer group between the primer region and the tails (not shown) which the polymerase cannot extend past and the tails remain single-stranded. vi) While the product of the first cycle is two duplexes each with one of the single-stranded tails, after multiple cycles the predominant product will be a duplex with both tails.

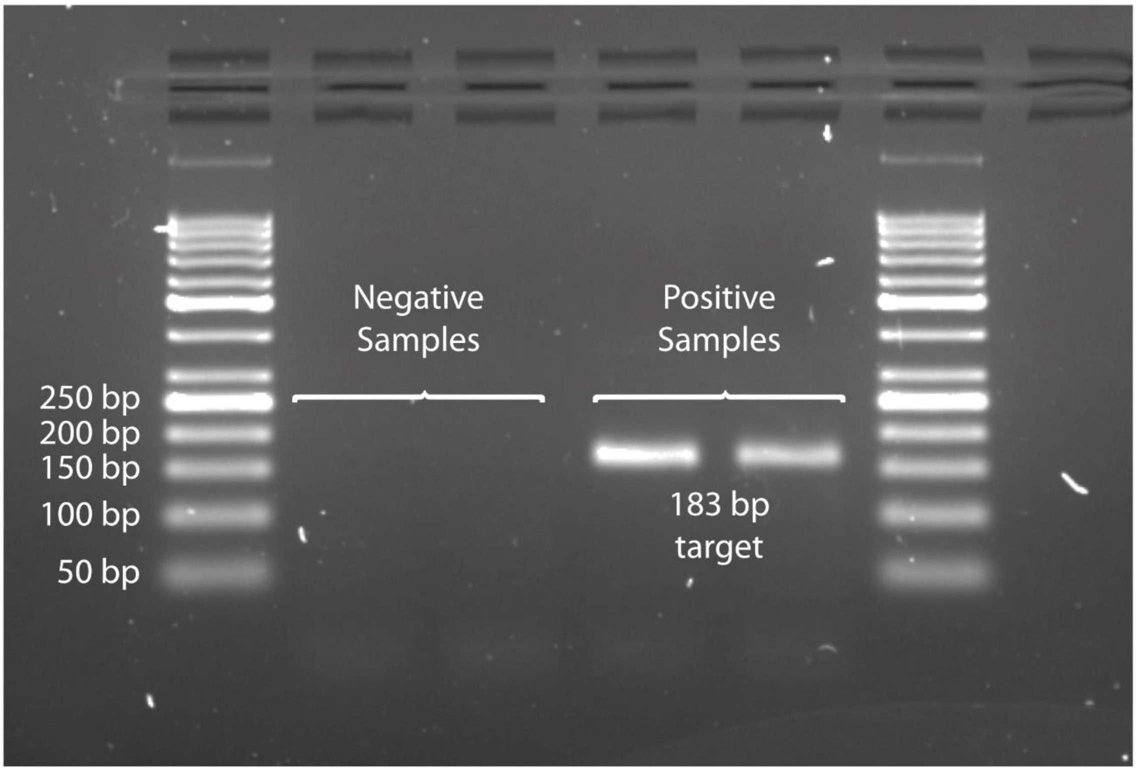

Figure S2. A characteristic example of an agarose gel electrophoresis of purified RPA product for two negative and two positive samples of *O.* cf. *siamensis*.

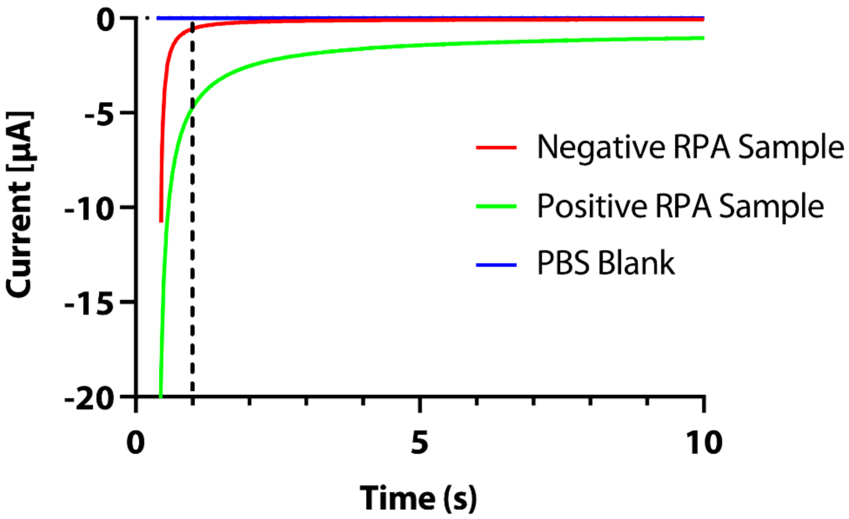

Figure S3. Representative plot of chronoamperometric measurement for positive and negative RPA samples, and a PBS blank, in which the RPA product/PBS mix was replaced by pure PBS, on a 4-well device (using *O.* cf *ovata* capture probes). The dashed line marks the chosen 1 s readout points where we estimate the largest difference between positive and negative samples to be.

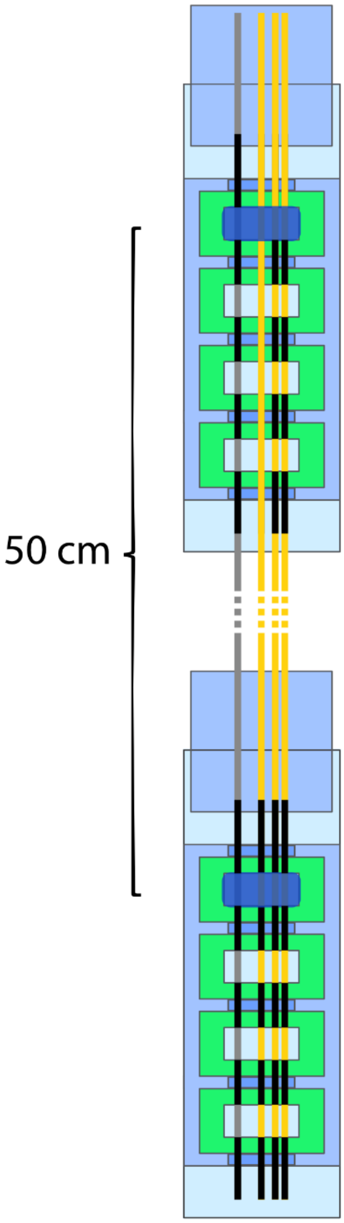

Figure S4. Schematic of two devices connected by long threads with two reaction regions spaced 50.0 cm apart. The lengths of the threads have been truncated for figure scaling purposes.

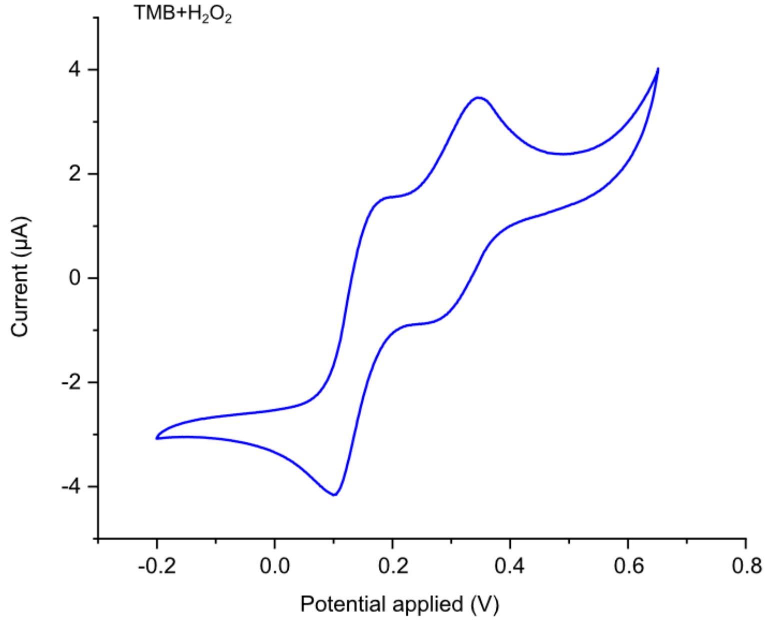

Figure S5. CV of Au threads in TMB and H_2O_2. The CV was obtained with an Autolab PGSTAT204N with MUX 16 module (Metrohm Autolab, Sweden) with the accompanying NOVA 1.11 software package.
